# Impact of extrapulmonary comorbidities on physical activity in chronic obstructive pulmonary disease in Japan: A cross-sectional study

**DOI:** 10.1371/journal.pone.0270836

**Published:** 2022-07-27

**Authors:** Makoto Yoshida, Tetsuya Hiramoto, Atsushi Moriwaki, Hisayuki Osoreda, Tomoaki Iwanaga, Hiromasa Inoue

**Affiliations:** 1 Division of Respiratory Medicine, National Hospital Organization Fukuoka Hospital, Fukuoka, Japan; 2 Division of Psychosomatic Medicine, National Hospital Organization Fukuoka Hospital, Fukuoka, Japan; 3 Department of Pulmonary Medicine, Graduate School of Medical and Dental Sciences, Kagoshima University, Kagoshima, Japan; Srebrnjak Children’s Hospital, CROATIA

## Abstract

Physical activity, which can be affected by airflow limitation and extrapulmonary comorbidities, has been reported to be reduced in patients with chronic obstructive pulmonary disease, and reduced physical activity is associated with higher risks of exacerbation and mortality. The aim of the present study is to elucidate the comprehensive effect of extrapulmonary comorbidities on physical activity in Japanese patients with chronic obstructive pulmonary disease, of which evidence is lacking. We conducted a cross-sectional study with a series of tests, including lung function, physical activity, symptom scores, and parameters for comorbidities. Sixty outpatients with stable disease were enrolled, and the relationship between the parameters and physical activity was evaluated. Physical activity was assessed over 7 consecutive days using a triaxial accelerometer, which records total daily energy expenditure, step count, and walking time. Cardiovascular status was assessed via echocardiography, and pulmonary artery pressure was estimated using Doppler sonography. As to mental status, depression and anxiety were assessed using the Self-Rating Depression Scale and State-Trait Anxiety Inventory, respectively. Physical activity level was significantly correlated with step count, walking time, body mass index, lean body mass index, lung function, pulmonary artery pressure, depression, anxiety, and serum total cholesterol level. In a median regression model analysis, low lung function, low lean body mass index, depression, and low serum total cholesterol level were independently associated with decreased physical activity level. These findings suggest that physical inactivity is affected by multiple extrapulmonary factors, including skeletal muscle dysfunction, depressive symptoms, and nutritional state, in Japanese patients with chronic obstructive pulmonary disease.

## Introduction

Chronic obstructive pulmonary disease (COPD) is characterised by persistent respiratory symptoms and airflow limitation that is due to airway and alveolar abnormalities usually caused by significant exposure to noxious gases [1]. Patients with COPD often experience shortness of breath or dyspnoea when they move, which makes them tire of moving and leads them to be more sedentary in their daily lives. Reduced physical activity causes further muscle weakness, which entraps them in a vicious downward spiral not only in their daily problems but also in the pathophysiology of the disease [2].

Exercise therapy in respiratory rehabilitation plays an important role in the management of COPD. Since exercise tolerance, or how much exercise a patient endures, is closely related to life prognosis in COPD [3], it has been used to evaluate athletic performance in respiratory rehabilitation. Recently, in addition to exercise tolerance, physical activity, which indicates how much the patient actually moves, has become recognised as one of the most important elements in the condition of COPD patients. Several observational studies have reported COPD patients with decreased physical activity disproportional to their restored exercise tolerance [4, 5].

Physical activity levels in CODP patients are reduced as compared to healthy adults without COPD, and such inactivity was observed even in Global Initiative for Chronic Obstructive Lung Disease (GOLD) stage I, the earliest stage of the disease [6–8]. Studies using an accelerometer showed that an increase in physical activity level was associated with a lower risk of hospitalisation and death. A level of physical activity equivalent to walking or cycling 2 hours/week or more was associated with a 30–40% reduction in the risks of hospitalisation due to COPD and respiratory mortality [9]. Furthermore, physical activity level is reported to be the best discriminative property for 4-year survival and was associated with the highest relative risk of death [10]. A systematic review has shown that physical activity affects not only hospitalisation and mortality but also COPD exacerbation, health-related quality of life, dyspnoea, and exercise tolerance [11]. Taken together, these results indicate that even in the early stage of the disease, COPD patients showed a decrease in physical activity, which will lead to multiple adverse outcomes, especially poor survival prognosis. Therefore, the importance of physical activity is emphasised in COPD guidelines [1, 12].

Several factors have been reported to affect physical activity in COPD. It is well known that airflow obstruction is correlated with physical inactivity in COPD [8, 13, 14]. Other studies have shown that systemic inflammation, skeletal muscle disorders, and depression are associated with decreased physical activity [13, 15, 16]. One study inclusively investigated factors that influence physical activity in COPD using multiple regression analysis, and the authors showed that systemic inflammation and left ventricular diastolic dysfunction are associated with decreased physical activity [17].

According to these reports, it is becoming clear that not only lung function but also extrapulmonary comorbidities can lead to the physical inactivity of COPD patients, which is associated with higher risks of exacerbation and mortality. However, studies investigating the comprehensive influence of extrapulmonary comorbidities on physical activity in COPD patients of Asian race are lacking. Therefore, we conducted a cross-sectional study with a series of tests assessing lung function, symptoms, and comorbidities to elucidate the factors affecting physical activity in Japanese patients with COPD. The results of the present study will provide new insights into comprehensive strategies for future management of COPD.

## Material and methods

### Study subjects and design

The study was performed in the outpatient clinic of the National Hospital Organization Fukuoka National Hospital from June 2014 through March 2015. Diagnosis of COPD was based on cigarette smoking history and postbronchodilator forced expiratory volume in 1 second (FEV_1_)/ forced vital capacity (FVC) < 0.70 according to the Global Initiative for Chronic Obstructive Lung Disease [1]. COPD subjects who were stable over the previous 4 weeks were recruited. We conducted a cross-sectional study with a series of tests assessing physical activity, lung function, and comorbidities. The minimum sample size was calculated to be 46 using following parameters; anticipated effect size (f^2^) as 0.35, desired statistical power level as 0.8, probability as 0.05 and number of predictors as 6. The present study was approved by the ethics committee of the National Hospital Organization Fukuoka National Hospital (approval number: 26–11), and each participant gave written informed consent.

### Physical activity measurement

We used a triaxial accelerometer, HJA-350IT (OMRON; Tokyo, Japan), which can record parameters including total daily energy expenditure, step count, walking time, and active time. Energy expenditure is expressed as Ex/day, which is calculated by multiplying metabolic equivalent of tasks (METs) by hours during the awake time of each day. Subjects wore a triaxial accelerometer for 7 consecutive days, and the mean values in each parameter were calculated.

### Lung function and assessment of comorbidities

Spirometry was measured with a Chestac-8900 (Chest Co. Ltd., Tokyo, Japan). The diffusing capacity of lung for carbon monoxide (*D*_LCO_) was measured using the single breath-hold method. To assess metabolic and muscular states, body mass index (BMI; body weight in kilograms divided by squared height in metres) and lean body mass index (LBMI; lean body weight in kilograms divided by squared height in metres) were determined. Echocardiography was performed to measure the left ventricular ejection fraction (LVEF) and estimated systolic pulmonary artery pressure (esPAP), which was calculated by adding the trans-tricuspid gradient and right arterial pressure as described in a previous study [18]. Depression and anxiety statuses were measured using the Self-Rating Depression Scale (SDS) and State-Trait Anxiety Inventory (STAI), respectively [19, 20].

In order to assess other comorbidities, serum levels of C-reactive protein (CRP), total cholesterol (TC), low-density lipoprotein cholesterol (LDL-C), albumin (Alb), triglyceride (TG), N-terminal pro-B-type natriuretic peptide (NT-proBNP), and blood glycated haemoglobin (haemoglobin A1c; HbA1c) were measured.

### Statistical analyses

Data analyses were performed using STATA version 14 (StataCorp LLC, College Station, Texas, USA). Differences among patients in each GOLD stage of COPD were assessed via analysis of variance (ANOVA) for normally distributed variables and the Kruskal–Wallis test for non-normally distributed variables. The univariate analysis was assessed using Spearman’s rank correlation coefficient. To determine whether each parameter independently affected physical activity, the physical activity data were divided into 10 groups to minimise the effect of bias, and multiple regression analyses were performed. The data were divided into groups in order to fit multiple regression analysis, because they were not normally distributed even after logarithmic transformation. Number of groups were set enough to fit multiple regression analysis and to divide the data equally in size.

## Results

### Subject characteristics

Sixty-six patients were recruited. Six subjects were excluded from analyses because their mean wearing time of the accelerometer was less than 6 hours/day. We conducted analyses in 60 patients, and their demographic and clinical characteristics are summarised in Tables 1 and 2. There was no significant difference in ages among each stage of GOLD. Physical activity level, step counts, walk minutes, BMI, and LBMI gradually decreased significantly with the GOLD stages. The HbA1c level was significantly different between GOLD stages 3 and 4 (post hoc comparisons using the Scheffe test).

**Table 1:** Mean values of demographic characteristics and pulmonary function by GOLD stages.

	Total	GOLD I	GOLD II	GOLD III	GOLD IV	P value
Gender, male/female	60 (52/8)	15 (10/5)	22 (19/3)	13 (13/0)	9 (9/0)	
Age (year)	73.7 (7.0)	72.8 (9.5)	74.9 (5.8)	75.0 (6.9)	70.1 (4.8)	0.222
Height (cm)	161.0 (6.6)	161.5 (8.0)	160.6 (6.8)	159.1 (5.1)	163.9 (5.7)	0.400
Weight (kg)	57.2 (10.9)	61.2 (11.8)	59.3 (9.8)	54.6 (11.3)	49.9 (8.4)	0.050
Physical activity (Ex/day)	1.38 (1.50)	2.13 (1.56)	1.60 (1.46)	0.99 (1.50)	0.15 (0.10)	<0.001
Step count (/day)	3293 (3002)	4876 (2973)	3687 (2954)	2507 (3016)	860 (785)	<0.001
Walking time (min/day)	52.2 (39.5)	68.4 (35.1)	58.7 (41.9)	46.8 (39.1)	16.8 (12.3)	0.002
BMI (kg/m^2^)	20.1 (3.88)	23.4 (4.22)	22.9 (3.20)	21.5 (3.92)	18.6 (3.15)	0.014
LBMI (kg/m^2^)	16.8 (2.09)	17.4 (1.60)	17.2 (1.81)	16.4 (2.81)	15.0 (1.33)	0.020
FVC (% predicted)	95.7 (21.7)	127.3 (12.8)	95.8 (11.1)	84.9 (15.4)	68.6 (15.9)	<0.001
FEV_1_ (% predicted)	62.2 (27.7)	99.3 (13.9)	63.4 (9.52)	47.6 (16.0)	22.9 (5.35)	<0.001
*D*_LCO_ (% predicted)	68.1 (29.6)	77.1 (19.6)	75.9 (30.5)	55.6 (33.6)	51.1 (25.5)	0.036

Values are given as the mean with standard deviations in brackets for continuous variables. A P value < 0.05 denotes statistical significance of the difference in each variable among GOLD stages.

GOLD, Global Initiative for Chronic Obstructive Lung Disease; BMI, body mass index; LBMI, lean body mass index; FVC, forced vital capacity; FEV_1_, forced expiratory volume in 1 second; *D*_LCO_, diffusing capacity of lung for carbon monoxide natriuretic peptide.

**Table 2:** Mean values of the parameters of extrapulmonary comorbidities by GOLD stages.

	Total	GOLD I	GOLD II	GOLD III	GOLD IV	P value
LVEF (%)	69.5 (6.13)	69.3 (4.50)	69.9 (7.29)	69.3 (5.48)	62.2 (7.07)	0.985
esPAP (mmHg)	34.0 (8.85)	32.8 (8.65)	33.3 (8.74)	34.0 (8.43)	37.9 (10.41)	0.550
SDS	38.9 (9.31)	38.0 (9.37)	36.2 (8.55)	42.2 (10.6)	42.9 (7.59)	0.147
STAI1	38.2 (9.89)	40.5 (11.27)	34.9 (7.42)	38.9 (9.89)	41.8 (12.0)	0.204
STAI2	39.0 (10.2)	39.9 (11.27)	36.9 (9.48)	40.7 (10.0)	41.9 (10.7)	0.453
NT-proBNP (pg/dL)	151.2 (208.2)	126.3 (152.5)	172.0 (289.9)	193.8 (145.4)	78.3 (69.5)	0.564
Alb (g/dL)	4.12 (0.37)	4.09 (0.31)	4.06 (0.41)	4.20 (0.40)	4.20 (0.32)	0.645
Hb (g/dL)	14.2 (1.29)	14.3 (1.28)	14.0 (1.16)	14.2 (1.70)	14.5 (1.10)	0.833
TG (mg/dL)	132.5 (81.6)	133.4 (110.5)	153.9 (87.9)	115.5 (39.1)	101.1 (36.8)	0.330
TC (mg/dL)	190.9 (34.9)	198.6 (38.6)	193.9 (31.71)	176.4 (32.9)	191.4 (38.5)	0.376
LDL-C (mg/dL)	102.2(29.2)	107.6(26.1)	107.4(30.2)	85.8(24.9)	103.9(32.9)	0.143
CRP (mg/dL)	0.24 (0.34)	0.13 (0.07)	0.21 (0.21)	0.36 (0.43)	0.34 (0.61)	0.224
HbA1c (g/dL)	5.84 (0.56)	5.86 (0.51)	5.78 (0.43)	6.16 (0.78)	5.5 (0.26)	0.040

Values are given as the mean with standard deviations in brackets for continuous variables. A P value < 0.05 denotes statistical significance of the difference in each variable among GOLD stages.

GOLD, Global Initiative for Chronic Obstructive Lung Disease; LVEF, left ventricular ejection fraction; esPAP, estimated systolic pulmonary artery pressure; SDS, Self-Rating Depression Scale; STAI = State-Trait Anxiety Inventory; NT-proBNP, N-terminal pro-B-type natriuretic peptide; Alb = albumin; Hb, haemoglobin; TG, triglyceride; TC = total cholesterol; LDL-C, low-density lipoprotein cholesterol; CRP, C-reactive protein; HbA1c, blood glycated haemoglobin

### Correlation between physical activity level and clinical parameters

As shown in Table 3, the results of univariate analysis showed that physical activity level was significantly correlated with the following parameters: step counts, walk minutes, BMI, LBMI, % predicted FVC, % predicted FEV_1_, % predicted *D*_LCO_, esPAP, SDS score, STAI1 score, and TC level.

**Table 3:** Relationship between physical activity and clinical characteristics assessed using Spearman’s rank correlation coefficient.

	r	P value
Age (years)	-0.122	0.374
Step count (/day)	0.920	<0.001
Walk time (minutes/day)	0.847	<0.001
BMI (kg/m^2^)	0.376	0.006
LBMI (kg/m^2^)	0.495	<0.001
FVC (% predicted)	0.540	<0.001
FEV_1_ (% predicted)	0.657	<0.001
*D*_LCO_ (% predicted)	0.514	<0.001
LVEF (%)	0.065	0.636
esPAP (mmHg)	-0.320	0.017
SDS	-0.457	<0.001
STAI1	-0.287	0.034
NT-proBNP (pg/dL)	-0.245	0.071
Alb (g/dL)	0.080	0.562
TG (mg/dL)	0.155	0.259
TC (mg/dL)	0.432	0.001
LDL-C (mg/dL)	0.341	0.012
CRP (mg/dL)	-0.139	0.318
HbA1c (g/dL)	0.033	0.811

BMI, body mass index; LBMI = lean body mass index; FVC, forced vital capacity; FEV_1_, forced expiratory volume in 1 second; *D*_LCO_, diffusing capacity of lung for carbon monoxide; LVEF, left ventricular ejection fraction; esPAP, estimated systolic pulmonary artery pressure; SDS, Self-Rating Depression Scale; STAI, State-Trait Anxiety Inventory; NT-proBNP, N-terminal pro-B-type natriuretic peptide; Alb, albumin; TC, total cholesterol; LDL-C, low-density lipoprotein cholesterol; CRP, C-reactive protein; HbA1c, blood glycated haemoglobin. The p-value associated with a correlation is a test of the null hypothesis that the correlation equals zero.

In order to elucidate the factors independently affecting physical activity, we conducted a multiple regression analysis, in which we adopted parameters to minimise confounding. We set the mean daily physical activity level to a dependent variable, and % predicted FEV_1_, LBMI, esPAP, SDS score, and TC level were selected as independent variables for multivariate analysis, in order to evaluate impact of airflow limitation and following comorbidities; skeletal muscle dysfunction, pulmonary hypertension, depression, and metabolic disorder. As indicated in Table 4, % predicted FEV_1_, LBMI, and TC level were independent variables of the increase in physical activity level, while the SDS score was an independent variable of the decrease in physical activity level.

**Table 4:** Relationship between physical activity and clinical parameters assessed via multiple regression analysis.

	β	t	P value
FEV1 (%predicted)	0.494	5.80	<0.001
esPAP (mmHg)	-0.004	-0.00	0.997
LBMI (kg/m2)	0.209	2.26	0.028
SDS	-0.252	-2.72	0.009
TCho (mg/dL)	0.280	3.12	0.003

Adjusted R^2^ = 0.621

FEV_1_, forced expiratory volume in 1 second; esPAP, estimated systolic pulmonary artery pressure; LBMI, lean body mass index; SDS, Self-Rating Depression Scale; TC, total cholesterol.

## Discussion

To our knowledge, this is the first study to report that multiple extrapulmonary comorbidities significantly affect physical inactivity in stable Japanese COPD patients independently of airflow limitation based on multivariate analysis. We showed that respiratory functions, LBMI, esPAP, SDS score, and TC level were significantly related to physical activity. Among these related parameters, LBMI, SDS score, and TC level were independent factors according to multivariate analysis with a median regression model.

Evidence of the inclusive effect of extrapulmonary comorbidities on physical activity in COPD patients is limited, with only a single comprehensive study using multiple regression analysis [17], in which the fibrinogen level, NT-proBNP level, and left ventricular diastolic dysfunction assessed by the deceleration time of the early transmitral flow and the systemic inflammation were reported to be extrapulmonary factors significantly affecting physical activity. On the other hand, in the present study, although esPAP as an index of right-sided heart function was significantly correlated with physical activity level in univariate analysis, multivariate analysis showed that this parameter was not an independent factor. The difference between the previous report and our result might be due to the difference in data processing, sample size, and sample profiles including race, as all enrolled subjects were exclusively Asian in the present study. As to data processing, we did not adopt age or gender in the regression analysis because that information is included in the % predicted FEV_1_ value.

In the present study, we found that LBMI had a stronger independent correlation with physical activity level than did BMI. Previous studies have shown that the fat-free mass index correlated with exercise capacity [21] and that higher muscle mass in lower limbs, assessed by analysing computed tomography (CT) scan images of bilateral mid-thighs, was associated with increased physical fitness and physical activity [15]. LBMI (kg/m^2^) is calculated by dividing squared height by lean body weight, which is highly dependent on muscle mass, as it is a subtraction of body fat from total body weight, including amount of muscle, bone, viscera, and water. Taken together, the correlation of LBMI to physical activity level can be assumed to be explained by that of muscle mass to physical activity. Moreover, LBMI is a more useful index than muscle mass assessment calculated based on CT image, because it is more convenient, less invasive, and more comprehensive, as it reflects the total muscle amount of the whole body.

Several mechanisms underlying the decrease in skeletal muscle mass independent of lung function in COPD patients have been reported, including systemic inflammation [22], inadequate nutritional intake [23], and reduced physical inactivity, which facilitates the decrease in muscle mass via disuse atrophy as a consequence. In turn, the decrease in muscle mass makes COPD patients less active by impairing exercise tolerance, leading to a vicious downward spiral of disease progression [2]. As a risk factor, the fat-free mass index is reported to be the third strongest predictor of high mortality after physical activity and airflow limitation [10]. Taking into consideration that skeletal muscle decreases independently of airway obstruction and that a decrease in muscle can lead to poor prognosis both directly and indirectly via decreasing physical activity, intensive intervention with comprehensive rehabilitation should be encouraged in COPD management for better outcomes [2].

Previous studies have shown that depression was negatively correlated with physical activity level and walking time in patients with COPD [16, 24], which is consistent with our results showing that the depression score was an independent factor with a negative influence on the physical activity level. It has been reported that depression identified in patients with stable COPD was significantly associated with higher risks of exacerbation and hospitalisation [25]. As physical activity level *per se* was reported to be an independent prognostic factor for mortality and hospitalisation in COPD patients [26], taken together with our result, depression is an important risk factor for poor prognosis among COPD patients via both direct influence and indirect influence through physical inactivity. Furthermore, it has been reported that COPD patients with a depressive mental status tend to be more sedentary after pulmonary rehabilitation than those without depression. Therefore, mental status can affect the therapeutic effects of rehabilitation [27], and intervention with respect to mental status will provide an important therapeutic strategy for the management of COPD [28].

To our knowledge, this is the first study to report a significant relationship between TC level and physical activity level in COPD patients. In order to explore the underlying mechanisms, we first analysed the correlation between physical activity level and each fraction of cholesterol, high-density lipoprotein cholesterol (HDL-C), and low-density lipoprotein cholesterol (LDL-C). There was no correlation between HDL-C level and physical activity level (r = 0.059, P = 0.514). Although the LDL-C level was significantly correlated with physical activity, its correlation coefficient (r = 0.340, P = 0.008) was smaller than that of the TC level (r = 0.432, P = 0.001). We next analysed the correlation between TC level and other clinical parameters to find a significant correlation with *D*_LCO_ (0.284, P = 0.035), esPAP (r = 0.462, P < 0.001), BMI (r = 0.299, P = 0.027), NT-proBNP (r = -0.361, P = 0.007), serum albumin (r = 0.418, P = 0.002), and haemoglobin (r = 0.309, P = 0.022). These results suggest that the TC level might be related to the physical activity level through cardiac function and nutritional status. Further studies are needed to elucidate the precise mechanism(s) underlying these findings.

The present study has some limitations. First, all subjects were enrolled from a single facility, and the sample size was relatively small. In order to verify the results of the present study and obtain more accurate information, a multicentre study with larger sample size is needed. Second, esPAP was used to assess pulmonary artery pressure. Although right heart catheterisation is apparently preferable for accurate evaluation, we did not adopt this procedure because of its invasiveness.

## Conclusion

In conclusion, low lung function, low LBMI, a high depression score, and a low TC level were independently associated with a decreased physical activity level in 60 Japanese patients with stable COPD in a median regression model analysis. These findings suggest that multiple extrapulmonary factors, including skeletal muscle dysfunction, depressive symptoms, and impaired nutritional state, lead to reduced physical inactivity and that intervention for these extrapulmonary comorbidities will provide new comprehensive strategies for better management of COPD.

## Supporting information

S1 Data (XLSX)
